# Transcriptome profiling data reveals ubiquitin-specific peptidase 9 knockdown effects

**DOI:** 10.1016/j.dib.2019.104130

**Published:** 2019-06-11

**Authors:** Enrico Glaab, Paul Antony, Sandra Köglsberger, Julia Ilona Forster, Maria Lorena Cordero-Maldonado, Alexander Crawford, Pierre Garcia, Manuel Buttini

**Affiliations:** a Luxembourg Centre for Systems Biomedicine (LCSB), University of Luxembourg, 7 avenue des Hauts Fourneaux, L-4362 Esch-sur-Alzette, Luxembourg; b Faculty of Veterinary Medicine, Norwegian University of Life Sciences, Oslo, Norway

**Keywords:** Transcriptomics, Microarray, Ubiquitin signaling, Knockdown, Cell culture

## Abstract

Ubiquitin specific peptidase 9 (USP9) is a deubiquitinase encoded by a sex-linked gene with a Y-chromosomal form (*USP9Y*) and an X-chromosomal form (*USP9X*) that escapes X-inactivation. Since *USP9* is a key regulatory gene with sex-linked expression in the human brain, the gene may be of interest for researchers studying molecular gender differences and ubiquitin signaling in the brain.

To assess the downstream effects of knocking down *USP9X* and *USP9Y* on a transcriptome-wide scale, we have conducted microarray profiling experiments using the human DU145 prostate cancer cell culture model, after confirming the robust expression of both *USP9X* and *USP9Y* in this model. By designing shRNA constructs for the specific knockdown of *USP9X* and the joint knockdown of *USP9X* and *USP9Y*, we have compared gene expression changes in both knockdowns to control conditions to infer potential shared and X- or Y-form specific alterations. Here, we provide details of the corresponding microarray profiling data, which has been deposited in the Gene Expression Omnibus database (GEO series accession number GSE79376). A biological interpretation of the data in the context of a potential involvement of *USP9* in Alzheimer's disease has previously been presented in Köglsberger et al. (2016). To facilitate the re-use and re-analysis of the data for other applications, e.g. the study of ubiquitin signaling and protein turnover control, and the regulation of molecular gender differences in the human brain and brain-related disorders, we provide a more in-depth discussion of the data properties, specifications and possible use cases.

**Table:** Specifications table

Subject area	*Biology*
More specific subject area	*Ubiquitin signaling*
Type of data	*Microarray gene expression data*
How data was acquired	*Gene expression profiling using Affymetrix Human Gene 2.0 ST arrays on an Affymetrix GeneChip Scanner 3000*
Data format	*Raw (CEL files) and preprocessed and normalized data using the GC-RMA approach*
Experimental factors	*Expression profiling was performed with triplicate samples for each of the following five considered conditions: USP9X/Y knockdown, USP9X knockdown, scrambled RNA control, transfection reagent control, and unperturbed control.*
Experimental features	*DU145 prostate cancer cells were transfected with shRNA constructs for the knockdown of USP9X/Y and USP9X knockdown using the Lipofectamine 2000 transfection reagent (control samples included a scrambled RNA control, a transfection reagent control, and an unperturbed control). Total RNA was extracted using the Qiagen RNeasy Mini Kit (Qiagen #74106) and treated with DNaseI (Qiagen #79254).*
Data source location	*Luxembourg Centre for Systems Biomedicine (LCSB), University of Luxembourg, 7 avenue des Hauts Fourneaux, L-4362 Esch-sur-Alzette, Luxembourg*
Data accessibility	*Data is accessible through the NCBI GEO database. The series record ID is GSE79376 located at*https://www.ncbi.nlm.nih.gov/geo/query/acc.cgi?acc=GSE79376.
Related research article	*Köglsberger S, Cordero-Maldonado MLL, Antony P, Forster JII, Garcia P, Buttini M, Crawford A, Glaab E (2016) Gender-specific expression of ubiquitin-specific peptidase 9 modulates tau expression and phosphorylation: possible implications for tauopathies. Mol Neurobiol 54:7979–7993.*

**Table:** 

**Value of the data** • Since USP9 has been implicated as a key sex-linked regulatory gene in neurodegenerative diseases, altering the degradation of alpha-synuclein in Parkinson's disease [1], [2], enhancing the toxicity of amyloid-beta 42 expression in a Drosophila Alzheimer's model [3], and modulating the gene expression of the Alzheimer's associated protein tau [4], the information provided in the data on USP9 knockdown effects may help researchers to study the specific functional roles for both the X- and Y-form of USP9 in the context of multiple complex human diseases. • USP9 is a prime example for a sex-linked gene with an X-form that escapes X-inactivation [5]. For researchers studying other gonosomal genes, the data could therefore provide a reference to test analysis methods to assess shared and differential knockdown effects for the X- and Y-form of sex-linked genes, and to compare the results with other genes. • Transcriptome-wide measurement data reflecting the downstream effects of knockdowns for key regulatory genes involved ubiquitin signaling and protein turnover control is scarce. Thus, the data presented here may serve both as a pilot dataset to prepare similar studies for other deubiquitinases and ubiquitinases and to compare the results across different studies.

## 1 Data

**Fig. 1:**
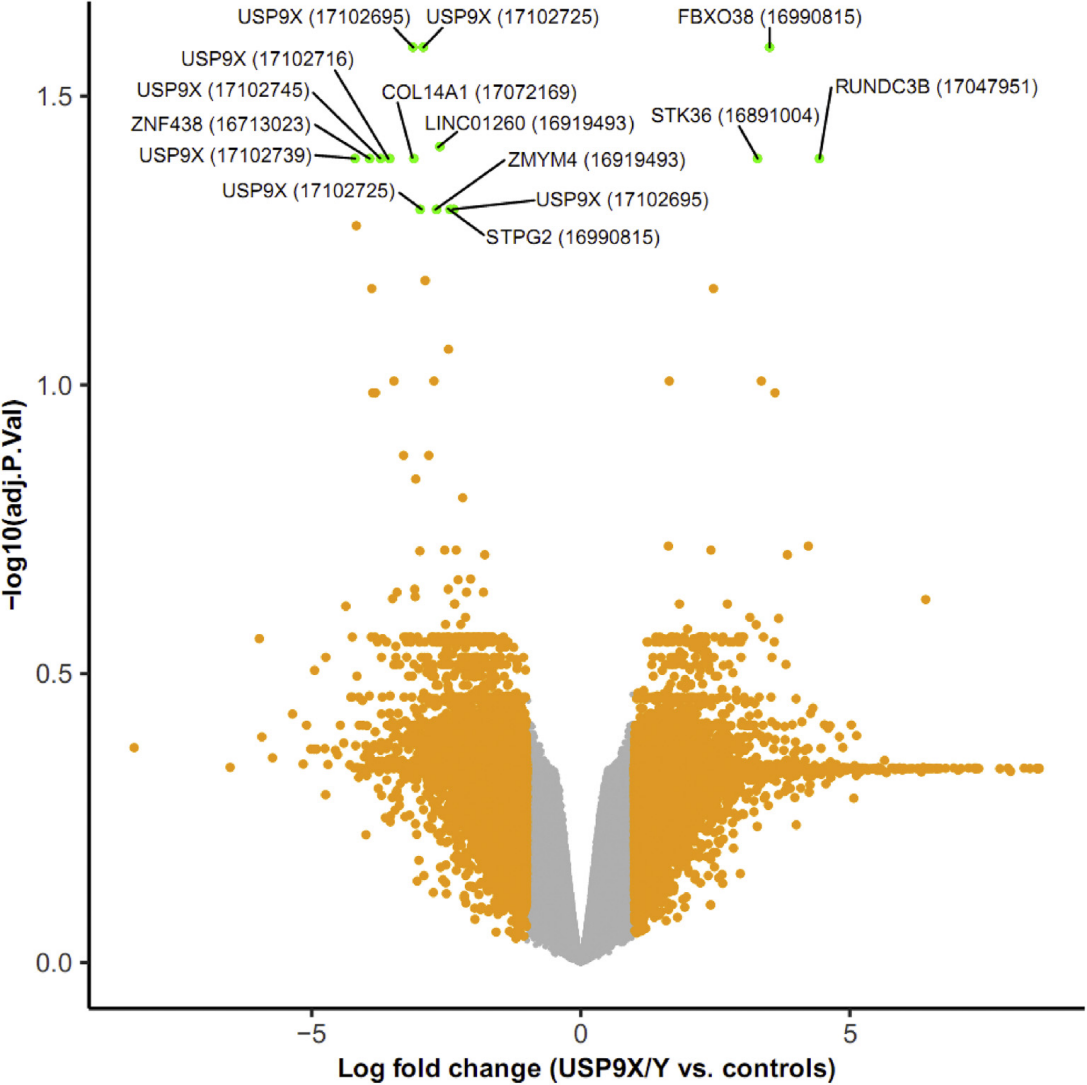
Volcano plot to visualize differential gene expression between *USP9X* and *USP9Y* combined knockdowns and control samples. For each transcript, the negative logarithm to base 10 for the adjusted p-value is plotted against the log fold change. To highlight transcripts with high effect size and significance, data points are colored orange if the absolute value of the log fold change is above 1, and green if additionally, the adjusted p-value is below 0.05 (all other data points are shown in black). For the data points that pass both these effect size and significance filters, the HGNC gene symbols and corresponding genetic probe set IDs (in brackets) are also displayed (multiple genetic probes map to the gene USP9X).

**Fig. 2:**
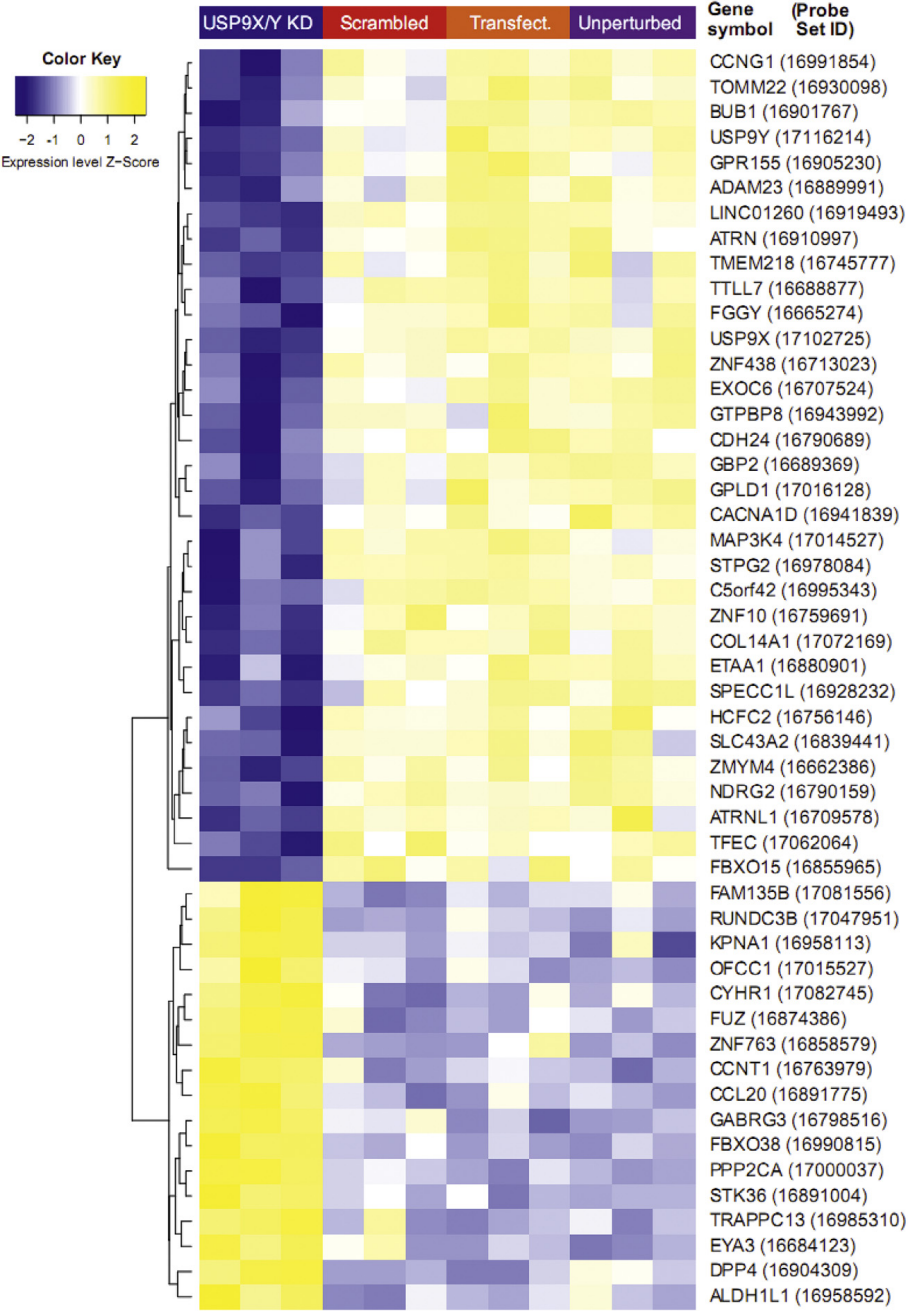
Heat map for the top 50 shared differentially expressed unique genes with known gene symbols in the USP9XY and USP9X knockdowns. On the right, the standard HGNC symbols for these genes are provided, as well as the IDs of the corresponding microarray genetic probe sets (shown in brackets). The heat map color codes represent row-wise Z-scores computed for the gene expression levels on log-scale (see the color key at the top left).

**Fig. 3:**
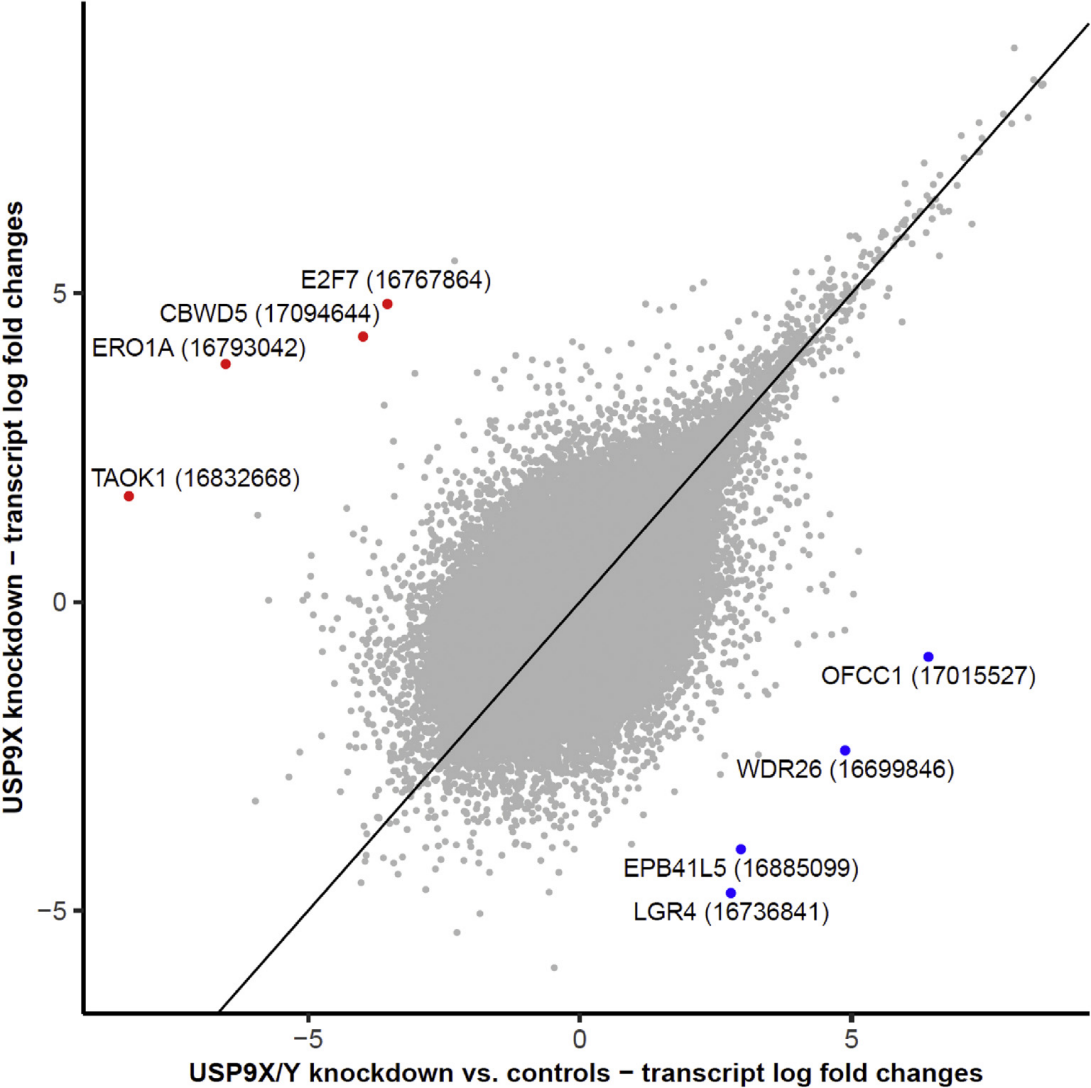
Correlation plot of gene expression log fold changes in the USP9X knockdown samples compared to controls vs. transcript log fold changes in the USP9X/Y knockdown samples. Each grey point corresponds to one genetic probe. The genetic probes with the largest differences in log fold changes between the two knockdowns are highlighted by bold colored points (red = higher log fold changes in the USP9X knockdown, red = higher log fold changes in the USP9X/Y knockdown) and the corresponding HGNC gene symbols and genetic probe IDs (in brackets) are displayed next to these points. The black diagonal line represents the identity line as a reference (slope = 1, intercept = 0).

Transcriptome-wide gene expression profiles of human DU145 prostate cancer cells were obtained for two different shRNA-knockdowns, one specific for the X-form of the gene *USP9* (*USP9X*) and one combined knockdown of both the X- and Y-form of the gene (*USP9X/Y*, used to infer potential *USP9Y*-specific gene expression changes indirectly by comparing both knockdowns). The data additionally covers transcriptome measurements for three control conditions to assess the significance and specificity of the knockdown effects: A scrambled RNA control, a transfection reagent control, and an unperturbed control. For each of these target and control conditions, three replicates are included to account for biological and technical variation in subsequent statistical analyses. Apart from the raw microarray measurements in Affymetrix CEL-file format, we also provide the pre-processed data obtained from applying the GC-RMA approach for background correction, normalization and probe replicate summarization [6]. To provide an overview of the significance and effect sizes of differentially expressed genes between USP9X and USP9Y combined knockdowns and control samples in this dataset, a volcano plot was generated (Fig. 1) and the top 50 differentially expressed unique genes with known gene symbols were visualized in a heat map (Fig. 2). Finally, to compare the expression changes in the two knockdowns against each other, a correlation plot of transcript log fold changes in the USP9X knockdown samples compared to controls vs. the transcript log fold changes in the USP9X/Y knockdown samples was created (Fig. 3).

## 2 Experimental design, materials and methods

### 2.1 Cell culture knockdown experiments

DU145 cells (ATCC #HTB-81) were cultured in Dulbecco's modified eagle medium (Gibco #41966–029), supplemented with 10% v/v heat-inactivated fetal bovine serum (Gibco #10500–064). Knock-down plasmids were designed to either jointly target *USP9X* and *USP9Y* or only target *USP9X*. Since *USP9X* and *USP9Y* have a high sequence homology, no potentially unspecific *USP9Y* shRNA plasmids were generated. Hairpin sequences and U6 promoters were subcloned into FastBac plasmids [7] (see [4] for details on the primers tested and the agarose gel electrophoresis results). Only one shRNA construct for each target was used, and since off-target effects can occur and the gene silencing efficiency may vary for different constructs, limitations may arise when interpreting data derived from a single construct. However, since the expression analysis only showed a highly significant inhibition of the target genes and no matched or similar effects for any other gene, we can exclude strong off-target changes and that larger amounts of target transcripts escape the inhibition to recover normal function. Cells were transfected via lipofectamine 2000 (ThermoFisher/Invitrogen #11668019) and incubated for 48 h.

### 2.2 Cell sorting, RNA isolation and microarray experiments

Before extracting RNA, the perturbed cells were enriched via fluorescence-activated cell sorting. The Qiagen RNeasy Mini Kit (Qiagen #74106) and DNaseI (Qiagen #79254) were used to extract and treat the RNA, respectively. Reverse transcription was done as described previously [8]. RNA extracts were prepared for the microarray experiments using the GeneChip WT PLUS Reagent Kit (Affymetrix, Manual P/N 703174 Rev. 2 and User Manual GeneChip Expression Wash, Stain and Scan for Cartridge Arrays P/N 702731 Rev. 4). A NanoDrop ND-100 (Thermo Scientific) and the 2100 Agilent Bioanalyzer (Agilent) were used to check RNA quality and integrity, respectively. The purified, sense-strand cDNA was fragmented by uracil-DNA glycosylase (UDG) and apurinic/apyrimidinic endonuclease 1 (APE 1) at the unnatural dUTP residues. The fragmented cDNA was labelled by terminal deoxynucleotidyl transferase (TdT) using the Affymetrix proprietary DNA Labelling Reagent covalently linked to biotin. 5.5 μg of single-stranded cDNA was used for fragmentation and labelling, and the GeneChip Hybridization, Wash and Stain Kit was used to hybridize and wash the cartridges. Control Oligonucleotide B2 and 20X Eukaryotic Hybridization Controls were added to the hybridization cocktail containing the labelled sample and injected into the cartridge. Incubation lasted 16 hrs. at 45 °C with rotation at 60 rpm. Next, the Fluidics Station 450/250 was used to wash and stain the Affymetrix GeneChip Human Gene 2.0 ST probe arrays. Using the Affymetrix GeneChip Scanner 3000, the probe arrays were then scanned after completion of the wash protocols.

### 2.3 Microarray data processing and visualization

The raw microarray dataset was pre-processed and normalized using the GC-RMA approach [6], and the raw and processed data have both been made available in the Gene Expression Omnibus (GEO) database (series accession number GSE79376). Differentially expressed genes between different knockdown conditions (*USP9X* only and *USP9X/Y* combined knockdown) and control conditions were assessed using the empirical Bayes moderated t-statistic [9]. To adjust the resulting p-values for multiple hypothesis testing the Benjamini–Hochberg procedure was applied [10]. Gene expression patterns were visualized using a volcano plot to highlight altered patterns of gene expression between *USP9X* and *USP9Y* combined knockdowns and control samples (Fig. 1). The volcano plot compares the negative logarithm to base 10 of the adjusted p-values against the log fold change, revealing that numerous genes display alterations with high effect size and high significance. As a complementary visualization, a heat map and cluster dendrogram was generated for the top 50 shared differentially expressed unique genes with known gene symbols in the *USP9XY* and *USP9X* knockdowns compared to control samples, using hierarchical average linkage agglomerative clustering with a Pearson correlation distance metric (Fig. 2). These top 50 genes include *USP9X* as the gene with the most significant decrease in expression levels. To further confirm the successful knockdowns for *USP9X* and *USP9Y/X*, we determined the median log fold changes and p-values for all genetic probes mapping to these genes. For the *USP9X/Y* knockdown, a strong under-expression was observed for both the *USP9X* and the *USP9Y* gene (median log. fold change (logFC) *USP9X*: -2.8, median p-value *USP9X*: 1.1E-05; median logFC *USP9Y*: -1.4, median p-value *USP9Y*: 6.1E-03). For the *USP9X* knockdown, lower average expression levels for *USP9X* were also observed, but with a weaker effect (median logFC: -0.46, median p-value: 2.6E-01). To assess the similarity between the expression alterations for the two knockdowns, a correlation plot was generated, comparing the transcript log fold changes for the USP9X knockdown against those in the USP9X/Y knockdown (Fig. 3).
